# Application of Low-Fermenting Yeast *Lachancea thermotolerans* for the Control of Toxigenic Fungi *Aspergillus parasiticus*, *Penicillium verrucosum* and *Fusarium graminearum* and Their Mycotoxins

**DOI:** 10.3390/toxins10060242

**Published:** 2018-06-14

**Authors:** Randa Zeidan, Zahoor Ul-Hassan, Roda Al-Thani, Virgilio Balmas, Samir Jaoua

**Affiliations:** 1 Department of Biological & Environmental Sciences, College of Arts and Sciences, Qatar University, P.O. Box 2713 Doha, Qatar; rz1604991@student.qu.edu.qa (R.Z.); zahoor@qu.edu.qa (Z.U.-H.); ralthani@qu.edu.qa (R.A.-T.); 2 Dipartimento di Agraria, Università degli Studi di Sassari, 07100 Sassari, Italy; balmas@uniss.it

**Keywords:** food safety, mycotoxins, biological control, yeast, decontamination, tomatoes

## Abstract

Mycotoxins are important contaminants of food and feed. In this study, low fermenting yeast (*Lachancea thermotolerans*) and its derivatives were applied against toxigenic fungi and their mycotoxins. *A. parasiticus*, *P. verrucosum* and *F. graminearum* and their mycotoxins were exposed to yeast volatile organic compounds (VOCs) and cells, respectively. VOCs reduced significantly the fungal growth (up to 48%) and the sporulation and mycotoxin synthesis (up to 96%). Very interestingly, it was shown that even 7 yeast colonies reduced *Fusarium’s* growth and the synthesis of its mycotoxin, deoxynivalenol (DON). Moreover, decreasing yeast nutrient concentrations did not affect the inhibition of fungal growth, but reduced DON synthesis. In addition, inactivated yeast cells were able to remove up to 82% of the ochratoxin A (OTA). As an application of these findings, the potentialities of the VOCs to protect tomatoes inoculated with *F. oxysporum* was explored and showed that while in the presence of VOCs, no growth was observed of *F. oxysporum* on the inoculated surface areas of tomatoes, in the absence of VOCs, *F. oxysporum* infection reached up to 76% of the tomatoes’ surface areas. These results demonstrate that the application of yeasts and their derivatives in the agriculture and food industry might be considered as a very promising and safe biocontrol approach for food contamination.

## 1. Introduction

Mycotoxins, the secondary fungal metabolites of toxigenic species are mainly produced by the genera *Aspergillus*, *Penicillium* and *Fusarium*. These toxigenic species predominantly contaminate cereals during pre- and post-harvest storage and many other stages [1]. The production of mycotoxins in grains depends on several factors, including humidity, temperature, water activity, mechanical damages and fungal toxigenic potentials [2]. Based on their toxicological profiles, aflatoxin B1 (AFB1), ochratoxin A (OTA), zearalenone (ZEN), fumonisins, T-2/HT-2 and deoxynivalenol (DON) have been recognized on the top of the list of more than 400 known mycotoxins [3].

The health issues in relation to mycotoxins led the food and feed control authorities to set regulatory limits to protect animal and human subjects from the toxic effects of these compounds. For each mycotoxin, based on its available toxicological data, region/country, type of commodity and intended use, the maximum limits are different. The European Union (EU) has set maximum limits of aflatoxins for cereal-based food as 2 µg/kg (AFB1) and 4 µg/kg (AF total), while in feed these limits are 20 µg/kg. Similarly, for OTA, these levels are of 3 µg/kg for food and 250 µg/kg for feed material, except for pigs and poultry [4].

The stability of mycotoxins during routine food and feed processing made clear the issues of mycotoxins and mycotoxicosis. This urged the scientists to search for a safer, environmentally friendly and broad-spectrum approach to counter the issues of mycotoxins and mycotoxicosis. So far, preventive strategies at pre-harvest (crop rotation, sowing date, resistant varieties etc.) and post-harvest (segregation, proper storage etc.) are in practice with variable success [5]. However, once the toxigenic fungi have infected the cereals and resulted in the accumulation of mycotoxins in it, the decontamination strategies then can be employed [6]. These procedures include chemical treatments (acids, basis, ozone, ammonia), physical treatments (adsorption, cooking, roasting, frying, baking and UV irradiation) and biological treatments (metabolic conversion, fermentation, enzymatic degradation) aiming to reduce the mycotoxins to safe limits [7,8,9]. In each case, along with other factors, the nature of target mycotoxin(s) and the pH of the medium determine the success of the employed technique [10].

Organic control using biological agents against mycotoxins is considered a safer option and is now getting popularity in the food industry [11]. In fact, the application of yeasts (cells and their volatiles) and yeast derivative have great potential to minimize the economic losses caused by mycotoxigenic fungi. Several yeast species have been identified to have effective fungal biocontrol activities where they can be utilized against toxigenic fungi to inhibit their growth and mycotoxins synthesis [12,13]. The biocontrol activities of antagonistic yeast against toxigenic fungi involve several mechanisms including space and nutrient competition, parasitism, biofilm formation, production of antifungal compounds and release of oxidants in the environment [14]. However, the fungal growth inhibition does not always predict the retardation of mycotoxins synthesis. Apart from this, some yeast species are known to have great potentials of binding mycotoxins on their cell wall surface [15,16], and others can degrade parent mycotoxin to less or non-toxic metabolites [17,18]. Above all the beneficial effects, the majority of yeasts species hold ‘Generally Considered As Safe (GRAS)’ status. In the present study, using three genera of toxigenic fungi, we aimed to investigate the effects of a low fermenting yeast (*Lachancea thermotolerans*) on their growth, mycotoxins synthesis and mycotoxins decontamination. Additionally, the volatile organic compounds released by yeast were tested for their preservation potential against phytopathogenic *F. oxysporum* infection in tomatoes. 

## 2. Results and Discussion

### 2.1. Biocontrol Activity of Yeast VOCs against Toxigenic Fungi

**Figure 1:**
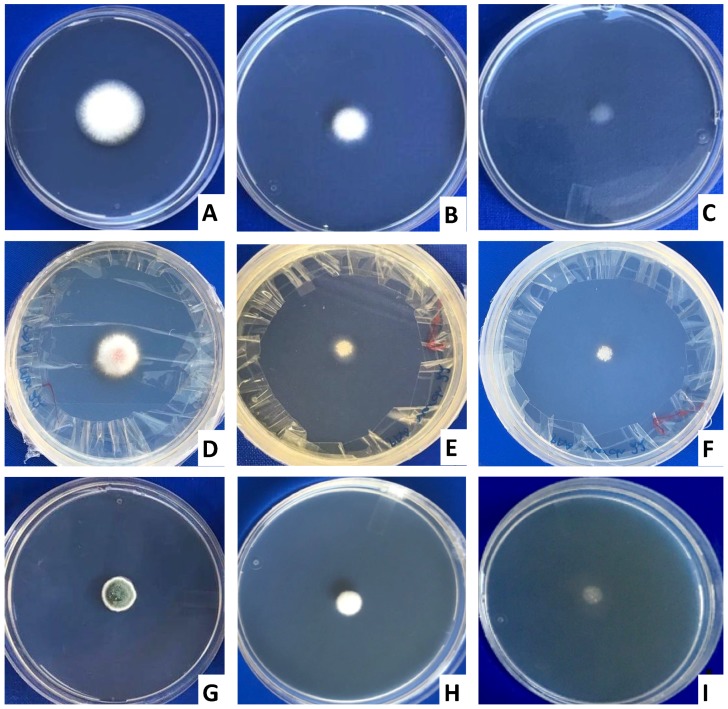
Biocontrol activity of yeast VOCs on *Aspergillus parasiticus* (**A**–**C**), *Fusarium graminearum* (**D**–**F**) and *Penicillium verrucosum* (**G**–**I**). Colony morphology of *A. parasiticus*; (**A**) not exposed to yeast VOCs; (**B**) exposed to VOCs from 100 yeast CFUs; (**C**), exposed to VOCs from 200 yeast CFUs. Colony morphology of *F. graminearum*; (**D**) not exposed to yeast VOCs; (**E**) exposed to VOCs from 100 yeast CFUs; (**F**), exposed to VOCs from 200 yeast CFUs. Colony morphology of *P. verrucosum*; (**G**) not exposed to yeast VOCs; (**H**) exposed to VOCs from 100 yeast CFUs; (**I**), exposed to VOCs from 200 yeast CFUs. In Figure 1 (**D**–**F**), presence of tapes at the margins shows how the sealing of two base plates opposite to each other was done.

**Figure 2:**
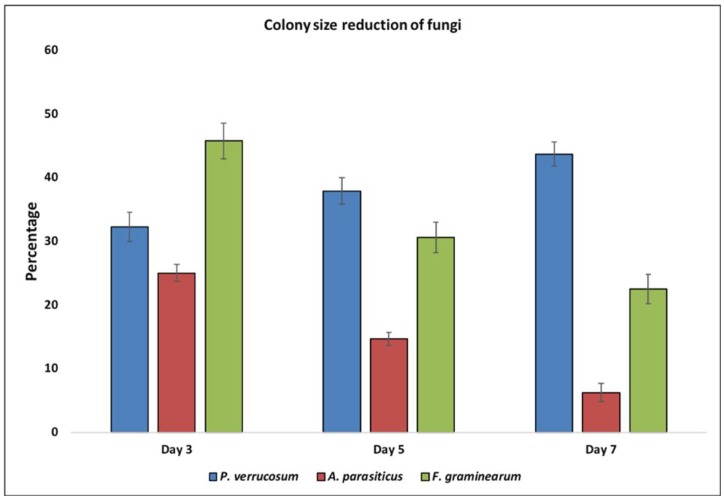
Fungal growth inhibition measured as colony size reduction (%) as compared to control at day 3, 5 and 7, of exposure to yeast VOCs. Spores of selected fungi were inoculated at the center of PDA plates and were sealed against already growing yeast colonies on YPDA.

At day 3 of post-exposure to yeast VOCs, *P. verrucosum* developed to a maximum colony diameter of 6.3 mm as compared to 9.3 mm of unexposed fungi. This showed an average 32% lesser development in fungal colony size as compared to the control (Figure 1 and Figure 2). With increasing the exposure time, the size of VOCs exposed fungus colony did not increase significantly, as compared to that of unexposed fungi. This led to increasing differences in colony diameter, as it was 9.5 mm and 10.3 mm on day 5 and 7, respectively, as compared to 15.3 and 18.3 mm in control. Colony size reduction was 37.91% (day 5) and 43.72% (day 7) as compared to control (unexposed fungi). Although not by the VOCs, the yeast isolated from the dry-cured hams in Italy, showed a similar inhibitory effect on the growth of *P. nordicum* [19]. These biocontrol activities of the yeast are assumed to be due to the release of toxic VOCs, as there was no physical contact between yeast and fungi.

In the present study, yeast VOCs also resulted in a significant decrease in *A. parasiticus* colony size, as compared to unexposed fungi. At day 3, 5 and 7 of exposure to yeast VOCs, colony diameter of *A. parasiticus* was 13.5 mm, 29 mm and 45 mm as compared to 18 mm, 34 mm and 48 mm in the control (unexposed to VOC), respectively. This decrease in colony diameter of VOCs exposed fungi, compared with the unexposed control was 25%, 15% and 6% at day 3, 5 and 7, respectively. These results demonstrated that increasing the yeast’s VOCs exposure duration to *A. parasiticus* results in the decrease in the severity of fungal growth inhibition, which might be the result of some resistant/tolerant phenomena in the fungus itself. The decrease in the colony size of *A. parasiticus* is likely to be associated with the production of 2-phenylethanol by yeast, which results in an alteration in the expression of the genes responsible for the fungal growth, as has been observed against *A. flavus* [20].

At day 3, 5 and 7, in response to yeast VOCs, *F. graminearum* showed an average colony diameter of 16 mm, 34 mm and 50 mm, which was significantly lower than 29 mm, 49 mm and 64 mm, respectively, as observed in (unexposed) control fungi. This decrease in colony size was 46%, 31% and 23% respectively at day 3, 5 and 7, as compared to unexposed fungi (Figure 2). In line with a present study, in field trials antagonistic yeast (*Cryptococcus nodaensis*) resulted in 50–60% reduction in *Fusarium* head blight in wheat caused by *F. graminearum* [21]. The fungal growth inhibition activities are likely to be associated with the yeast VOCs having 2-phenylethanol, that is a major anti-fungal compound [22,23].

### 2.2. Effect of Yeast VOCs on the Mycotoxins Synthesis

**Figure 3:**
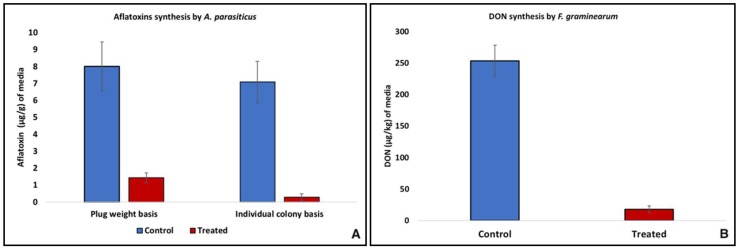
Inhibition of mycotoxins synthesis by toxigenic *A. parasiticus* and *F. graminearum* exposed to yeast VOCs; (**A**) comparison of aflatoxins synthesis between VOCs exposed and unexposed *A. parasiticus* on plug weight and individual colony basis; (**B**) Comparison of DON synthesis between yeast VOCs exposed and unexposed *F. graminearum*.

The production of mycotoxins by toxigenic fungi, that comes as a result of the reduction of the active fungal growth, leads to the upregulation of the toxigenic genes, as studied in *A. flavus* [23]. In the present study, exposure to yeast VOCs, resulted in a significant reduction in aflatoxins (AFs) synthesis by *A. parasiticus*. In the colonized media plugs, obtained from the yeast VOCs exposed fungi, significantly lower AFs contents (1.43 μg/kg) were noted, as compared to those obtained from unexposed fungi (8.01 μg/kg). This accounted an overall 82% decrease in AFs synthesis by yeast VOCs exposed fungi, compared to the control. A 96% lower AFs synthesis was noted in a yeast VOCs exposed *A. parasiticus* fungal colony, developed from a single spore, as compared to unexposed fungal spore colony (Figure 3). This demonstrated a precise approach to record the effect of yeast VOCs on mono-spore fungal mass. In a similar approach, the addition of 50 µL/L of gaseous allyl isothiocyanate in the fungal growth media resulted in the inhibition of *Aspergillus parasiticus* growth at 3.17 log CFU/g as compared to the control [24]. In line with the present study, inoculation of a baker’s yeast in the liquid media having *A. flavus* spore suspension resulted in 15.4 to 41.6% decreased AFB1 synthesis [25]. The suppression of mycotoxins synthesis by the toxigenic fungi, exposed to yeasts, principally happens to be the function of yeast metabolites, mainly 2-phenylethanol, which inhibits fungus growth and/or suppresses the genes involved in mycotoxins synthesis [21].

In case of *F. graminearum* exposed to yeast VOCs, in the colonized media plugs, the DON contents were 18.29 µg/kg as compared to the control (unexposed to VOCs), where it was 254 µg/kg. This showed an overall 93% lower DON synthesis in exposed fungi compared to the unexposed. In line with the findings of a present study, *Saccharomyces cerevisiae* did not only significantly inhibit the growth of toxigenic *F. graminearum*, but also resulted in the decrease of DON and ZEN synthesis [26].

### 2.3. Effect of No. of Yeast CFU on Fungal Growth and Mycotoxins Synthesis

**Figure 4:**
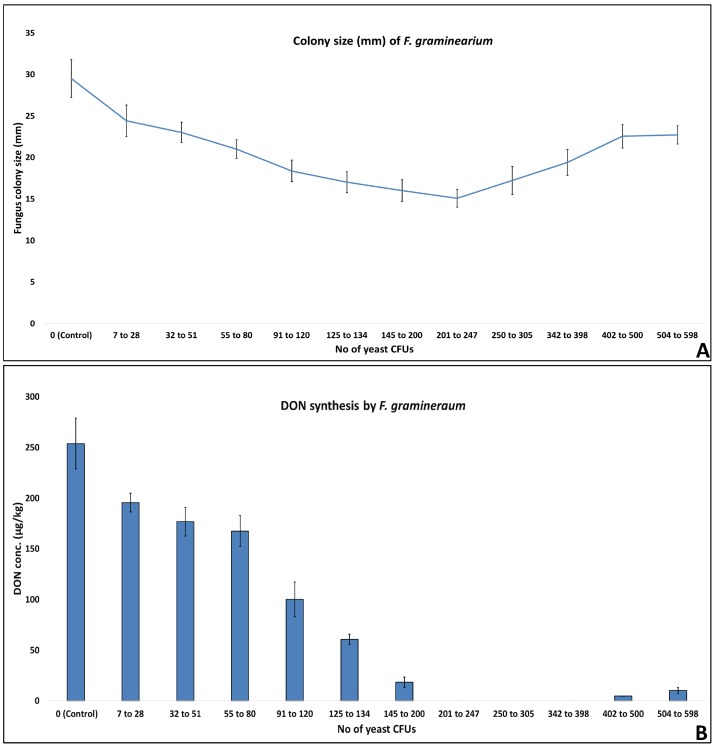
Kinetics of colony size and DON synthesis by *F. graminearum* in response to exposure to increasing numbers of yeast CFUs. (**A**) The colony size of fungi showing a decreasing trend upon exposure to yeast VOCs released from 7 to 250 yeast CFUs, afterwards the effect was reversed; (**B**) Mycotoxins synthesis by fungi showing a decreasing trend upon exposure to yeast VOCs released from 7 to 250 yeast CFUs, afterwards the effect was reversed, an almost similar trend to colony size observed in (**A**).

A curve was observed in *F. graminearum* colony size and DON synthesis in response to exposure to VOCs from increasing numbers of yeast CFUs. VOCs released from as low as 7 yeast CFUs showed an antagonist effect on *F. graminearum* colony size and DON synthesis. A gradual decrease in fungal colony diameter was noted with increasing yeast VOCs up to 247. Increasing the number of yeast CFUs from 250 and onwards, resulted in an increase in fungus colony diameter. A similar trend in the DON synthesis was observed in response to increasing yeast CFUs. The exposure of fungi to VOCs released from 7 yeast CFUs and up till 200, exhibited a gradual decrease in DON synthesis. The VOCs generated from 201 to 398 yeast CFUs, resulted in a complete inhibition of DON synthesis by toxigenic *F. graminearum*. Increasing the yeast CFUs from 402 again resulted in DON synthesis by *F. graminearum* (Figure 4). In the published literature, no such report exists regarding the kinetics of fungal growth and toxin synthesis in response to increasing the amount/number of yeast cells/VOCS. However, it is known that yeast or their VOCs inhibit the growth and mycotoxins synthesis of *F. graminearum* [21,26]. In the first study of its kind, we are able to conclude that to achieve maximum antagonistic effect on fungal growth and DON synthesis, 200–250 yeast CFUs produce optimum VOCs, in 90 mm petri plate experimental settings.

### 2.4. Role of Nutrients Availability to Yeast on Its Antagonistic Activities against F. graminearum

The findings noted in Section 2.3, guided us to explore the role of nutrients availability to antagonistic yeast on its antifungal activities. Three concentrations of the Yeast Extract Peptone Agar (YPDA); 1×, 0.5× and 0.1× were used to inoculate yeast cells. A significant reduction in the colony diameter of *F. graminearum* was noted upon exposure to yeast VOCs from 1× and 0.5× YPDA, while the effect was the opposite when yeast was grown on 0.1× YPDA. However, an inverse relation between nutrients availability to antagonistic yeast and DON synthesis by the toxigenic fungi was noted (Table 1).

A non-significant effect on the colony diameters and a significant reduction in the DON synthesis alongside with decreasing the nutrient availability to antagonistic yeast, is conceivably thought to be due to down-regulation of mycotoxins biosynthetic genes with no effect on the genes responsible for fungal growth. The effect of the yeast’s (*Pichia anomala*) VOCs upon growth- and toxigenic gene expression in *A. flavus* showed a many-fold down regulation [20]. However, the findings of the present study are in contrast with those of Reference [21], who by using *Cryptococcus* isolates, reported 50–60% lesser severity of *Fusarium* head blight caused by *F. graminearum.* These differences in VOCs exposed fungal growth and mycotoxins synthesis indicate yeast- and fungus- species-specific antagonistic mechanisms. Further exploration in these directions may provide a valuable explanation of the mechanism involved.

### 2.5. Mycotoxins Binding onto Yeast Cell Wall (YCW)

Application of yeast cells and their derivatives as mycotoxins decontaminating agents in food and feed industry have been widely reported [27,28,29]. The efficacy of yeast-based mycotoxins binding agents is potentially related to the nature of targeted mycotoxins, their polarity, the pH of the medium, contact duration and the nature of the yeast itself [30]. Considering these variables enhance the mycotoxins binding spectrums, most of the commercially available mycotoxins binding products contain mixture of yeast, bacterial enzymes and some clay. Adding live or inactivated yeast cells in the buffer solution, already contaminated with various levels of OTA, resulted in a significant removal of the toxin from the solutions. At an initial contamination level of 0.9 µg/kg, live yeast cells were able to reduce OTA at 63% and 67% in the supernatant of buffer solution of pH 5 and pH 7, respectively. However, in the pellet of pH 5 and pH 7 buffer solutions, 29% and 30% of the OTA was detected. The differences in the OTA contents (those removed from the supernatant and found in the pellet) may be caused by the degradation of the toxin by live yeast or its enzymes. The adsorption of OTA on the YCW has been reported to be a function of pH of the media as well as thickness of the YCW itself [10,26]. 

**Figure 5:**
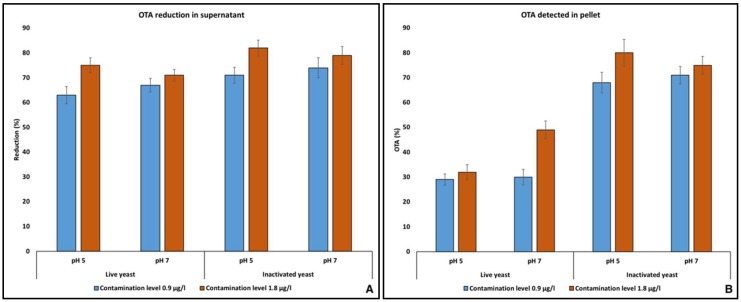
Adsorption of OTA by live and inactivated yeast cells; (**A**) percentage of OTA removed from the supernatant of the buffer solutions; (**B**) percentage of the OTA detected in the pellet.

At higher OTA contamination levels (1.8 µg/kg), live yeast cells showed mycotoxin adsorption/reduction in the supernatant at 75% and 71% in the buffer solution of pH 5 and pH 7, respectively. However, unexpectedly, in the pellet of these solutions, 32% (pH 5 buffer) and 49% (pH 7 buffer) of the total OTA was found. In the case of inactivated yeast, the amount of OTA removed from the supernatant of buffer solutions was higher, and its significant quantity was detected in the pellets of corresponding buffer solutions. The reduction of OTA in the supernatant of buffer solutions of pH 5 and 7 were 71% and 82%, respectively (Figure 5). Almost all amount of OTA removed from the supernatant was found in the pellet (68% and 80% respectively in pellet of pH 5 and pH 7 buffer). By exploring the interaction between some mycotoxins and yeast cell wall components, it was identified that there exists weak hydrogen and van der Waals bonds making the interaction more “adsorption” rather than “binding” [31]. The interaction of OTA and YCW involves polar as well as non-polar (hydrophobic amino acids of YCW and aromatic ring of OTA) bindings followed by rearrangement of the water in the solvent [32].

In the case of AFs, the adsorption of toxins on YCW was lower as compared to OTA. At pH 5, at contamination levels of 0.2 µg/kg and 0.4 µg/kg, the adsorption of AFs on live yeast cells was 16% and 34%, respectively. In the buffer solutions contaminated with AFs at 0.2 and 0.4 µg/kg, the addition of inactivated yeast cells resulted in 20% and 29% removal of the toxins. At pH 7, adsorption of AFs was much lower (up to 10%) by the live and inactivated yeast cells. In line with the present study, lower adsorption of AFB1 at pH 7, compared to pH 5, and overall lesser adsorption (10%) was noted by adding 5 mg of YCW power in buffer solution [10]. In another study, the adsorption of AFB1 on YCW was lower as compared to OTA and ZEA [33], as has been observed in the present study.

In a similar experimental setup, live yeast cells showed a significant lower DON adsorption (17%) from a buffer solution of pH 5 when compared to that at pH 7, which was 52%. However, in the pellet of live cells, DON was not detected at all, which can be explained by the fact that it might have been degraded into metabolites by live yeast cells. More detailed studies are needed to explore the possible mechanism of DON removal by live yeast cells. By the inactivated yeast, less significant removal of DON from the supernatant was observed, while significant concentration of the removed toxin was detected in the pellet (data not shown). In line with the present study, adsorption of DON on yeast was lesser (12.6%) as compared to other mycotoxins, like ZEN (66.7%), fumonisins (67%) and T-2 toxin (33%) [30]. 

### 2.6. VOCs Inhibits F. oxysporum Infection in Tomato

**Figure 6:**
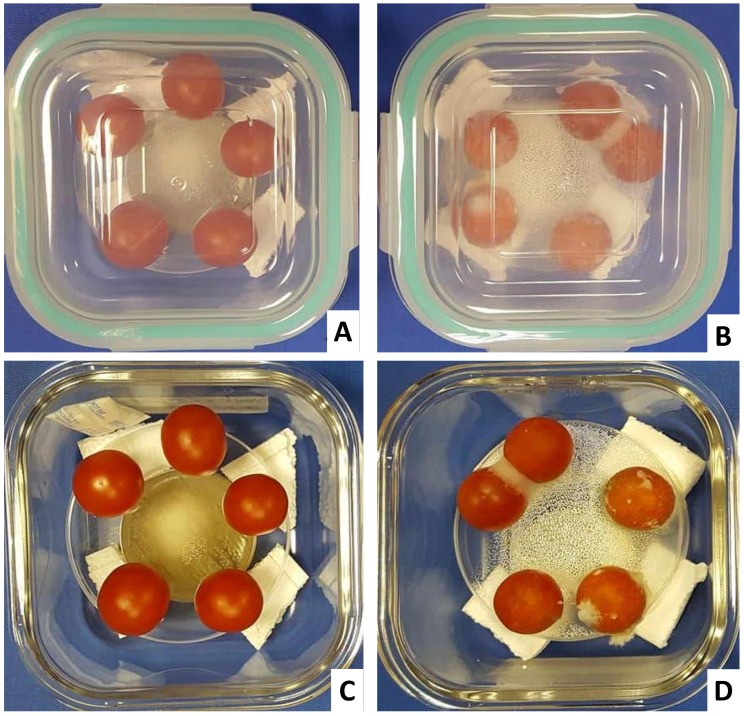
In vitro biocontrol activity of yeast VOCs against *F. oxysporum* infection on tomato; (**A**) tomato inoculated with *F. oxysporum* on their surface and exposed to yeast VOCs, from a streak inoculated 60 mm Petri dish with live yeast cells; (**B**) tomatoes inoculated with F. oxysporum, but not exposed to yeast VOCs. Tomatoes after 34 days of incubation at 26 °C; (**C**) tomato incubated in the presence of yeast VOCs showing no fungal growth; (**D**) tomato incubated without VOCs showing extensive fungal growth on the surface.

Surface inoculated tomatoes with *F. oxysporum* showed development of fungal infection, which spread to wider surface areas (Figure 6). The infection rate was scored on the basis of visible mycelium mass on the tomatoes’ surface area and it showed an average contamination of 76% (Figure 6).

On the other hand, tomatoes having inoculated fungal spores on their surface and exposed to yeast VOCs showed complete absence of infection till the end of the experiment (day 34 post- infection). These findings proved that VOCs were able to completely inhibit the fungal growth and germination, and hold the potential to protect the vegetables (tomatoes) from fungal infection for a longer time. In lines with present study, yeast cells sprayed on detached grape barriers were able to be protected from the fungal infection by *A. carbonarius* [22].

## 3. Conclusions

Biocontrol activity of low fermenting yeast (*Lachancea thermotolerans*) was tested against three fungi, each belonging to a different genus. The VOCs produced by antagonistic yeast inhibited the growth and sporulation of all three fungi as compared to the control. AF synthesis by *A. parasiticus* and DON synthesis by *F. graminearum* were significantly reduced by yeast VOCs. The effect of yeast VOCs on *F. graminearum* growth and DON synthesis was noticed from as low as 7 yeast CFUs, and showed a direct relation up to ~200 CFUs and then after a stationary phase (~450 CFUs) the effect was reversed. Reducing the availabilities of nutrients (as monitored by using different media dilutions) resulted in a non-significant effect on the growth of toxigenic *F. graminearum*; however, DON synthesis was significantly reduced. Live and inactivated yeast cells were able to remove 71% and 82% of OTA from the buffer solutions, respectively. Finally, in the most applied part of this work, using tomatoes artificially inoculated with *F. oxysporum* spores for 34 days it was demonstrated that yeast VOCs could inhibit the growth of *F. oxysporum* spores completely; while in the control exposed to no yeast cells, the infection rate scored on the basis of visible mycelium mass on tomatoes surface showed an average contamination of 76%. Therefore, it can be postulated that low-fermenting yeast (*Lachancea thermotolerans*) holds a strong potential as a biocontrol, decontaminant and preservative agent against significant toxigenic fungi and their mycotoxins.

## 4. Materials and Methods

### 4.1. Chemicals, Supplies and Biological Strains

Aflatoxins (B1, B2, G1, G2), OTA and DON powders were obtained from Romer Labs. ELISA kits for aflatoxins (RIDASCREEN^®^ Aflatoxin Total, Art No. R4701), OTA (RIDASCREEN^®^ Ochratoxin A, Art No. R1311) and DON (RIDASCREEN DON, Art No. R5906) were purchased from R-Biopharm AG, Darmstadt, Germany. The buffer solutions of pH 5 (acetate buffer) and pH 7 (phosphate buffer) were prepared according to the protocol described by Faucet-Marquis et al. [10]. ELISA plate reader (Multiskan FC, Thermo Scientific, Waltham, MA, USA) installed with Skanlt software (Version 4.1. Thermo Scientific, MA, USA, 2015) was used to obtain the absorbance of mycotoxins in ELISA plates. A calibration curve was generated by using absorbance data of known mycotoxins’ standards solutions (5–6), and the absorbance values of unknown samples were added to the calibration curve to calculate the amount of toxins in our samples. For this purpose, a software (Z9996 RIDA^®^-SOFT Win, R-Biopharm, Darmstadt, Germany) was used. Low fermenting yeast (*Lachancea thermotolerans* 751) was obtained from Dipartimento di Agraria, Universita di Sassari, Italy. *A. parasiticus*, *P. verrucosum* and *F. graminearum* were isolated from animal feed samples obtained from an animal feed market located in Doha, Qatar. Morphological examination was followed by PCR based identification of these isolates. Mycotoxins synthesis potential of the isolates was confirmed by the presence of key genes involved in biosynthetic pathways followed by in vitro analysis of the production of mycotoxins on laboratory media [34].

### 4.2. Effect of Yeast VOCs on Growth and Sporulation of Toxigenic Fungi

To ascertain the effect of yeast VOCs on the growth and sporulation of *A. parasiticus*, *P. verrucosum* and *F. graminearum*, yeast and fungal co-culture experiments were performed [22]. The experimental setup prevented the direct contact of yeast with fungal colony. Yeast extract peptone dextrose agar (Yeast extract: 10 g, peptone: 20 g, dextrose: 20 g and agar: 15 g for 1 L of media) plates were streak inoculated with 100 µL of yeast cell suspensions (10^6^ cells/mL) and incubated for 48 h at 25 °C. The lid of the plate was replaced by another Petri plate that was point inoculated with 10 µL of either *A. parasiticus* (10^7^ spores/mL), *P. verrucosum* (10^6^ spores/mL) or *F. graminearum* (10^4^ spores/mL). These plates were tightly sealed with parafilm^®^ with additional layers of adhesive tape to block the leakage of VOCs. The control plates of fungi were sealed with YPDA plates without yeast cells. The colony characteristics (size, sporulation and colony morphology) were measured at days 3, 5 and 7 post-sealing.

### 4.3. Effect of Yeast VOCs on the Mycotoxins Synthesis

In the experiments above (Section 4.2), on day 10 (*Aspergillus parasiticus*) and day 15 (*F. graminearum*), three plugs (6 mm each) from the colonized media were removed with a cork borer and then weighed. Mycotoxins were extracted in 1 mL of 70% methanol and sonicated for 60 min. A total of 500 µL of the extract was transferred to a new vial and was allowed to dry under a stream of Nitrogen. The dried extract was re-suspended in 500 µL of 10% methanol (for aflatoxins) or distilled water (for DON) before performing ELISA. 

### 4.4. Effect of Number of Yeast Colony Forming Units (CFU) on F. graminearum Growth and DON Synthesis

To record the effect of variable numbers of yeast CFU on the fungal growth and mycotoxin production, 100 µL of serial dilutions of yeast cells suspensions (20 dilutions) were spread inoculated on the YPDA plates. After 48 h of the yeast cells growth, these plates were sealed against *F. graminearum* point inoculated (10 µL from 10^6^ spores/mL) PDA plates. The effect of as low as 7 CFUs up to 598 yeast CFUs on the *F. graminearum* growth zone was recorded. Diameters of developing fungal zones were recorded at day 3, and the DON synthesis was confirmed at day 21 of the experiment. For each yeast condition or dilution, at least 3 replicates were performed. The sealing of fungal spores inoculated plates against yeast plates was performed as described above.

### 4.5. Effect of Nutrient Availability to Yeast on Its Antagonistic Activities

In order to test the role of nutrient availability on the antagonistic spectrum of yeast, three YPDA medium concentrations; 1× (as described in Section 4.2), 0.5× and 0.1× were used. Live yeast cells from overnight culture in YPDB were plated on each medium plate. Three media plates, each from different concentrations of YPDA having almost same number of yeast CFUs, were selected for sealing against *F. graminearum*. At day 3 of post-sealing, the diameter of developing colony was measured whereas, at day 21, DON synthesis was determined as described above.

### 4.6. In-Vitro Mycotoxins Binding Experiments

For the preparation of inactivated yeast, overnight yeast cells culture in YPDB (yeast extract: 10 g, peptone: 20 g, dextrose: 20 g in 1 L of distilled water) were maintained in a shaking incubator at 26 °C. After autoclaving and centrifugation briefly at 5000× *g*, the supernatant was discarded and the pellet was re-suspended in distilled water. The contents were centrifuged at 5000× *g* for 5 min at 4 °C. The residues were dried at 80 °C for 12 h before grinding them into powder form [35]. A total of 5 mg of YCW or 20 µL of live yeast culture was incubated in 990 µL or 970 µL of each buffer for 5 min, respectively. Mycotoxin solutions (10 µL) were added to each tube to reach the final concentration of 0.9 and 1.8 µg/kg of OTA, 0.2 and 0.4 µg/kg of AF and 40 and 80 µg/kg of DON. Tubes were incubated at 37 °C with end to end continuous shaking for 30 min. Supernatants were separated by centrifugation at 9200× *g* and collected in new tubes. After drying using SpeedVac, the pellets were re-suspended in 0.13 M sodium hydrogen carbonate solution, 10% methanol or distilled water for analysis of each mycotoxins; OTA, AF and DON. ELISA assays were performed for each supernatant and pellet.

### 4.7. In-Vitro Testing of Yeast VOCs against F. oxysporum Infection in Tomato

Organic tomatoes produced locally in Qatar were purchased from the supermarket. In disinfected glass containers (12.5 × 12.5 × 5.5), five tomatoes of similar weight (8 ± 0.45 g) were placed on a sterile platform. Below the tomatoes, a Petri dish (60 × 15 mm) having a full surface growth of yeast cells was placed in a way to allow the VOCs to disperse throughout the box. On the surface of each tomato, 5 µL of *F. oxysporum* cell suspension (10^4^ spores/mL) was placed. After sealing, boxes were shifted to an incubator at 26 °C for 34 days. In the control boxes, YPDA plates non-inoculated with yeast cells were placed. An objective scoring of fungal infection on the surface of each tomato was recorded.

### 4.8. Statistical Analysis

The obtained data was subjected to analysis of variance test (ANOVA). The mean values of treated groups were compared with the untreated control by using *t*-test. The means were considered as significant at *p* ≤ 0.05. SPSS statistical software (Version 23, IBM, NY, USA, 2017) was used for this purpose.

## Figures and Tables

**Table 1:** Effect of nutrient availability to yeast on its antagonistic activities.

Treatment	Colony Diameter (mm)	DON Synthesis (µg/kg)	Colony Diameter and DON Synthesis by Fungi Not Exposed to Yeast VOCs	
Control (No yeast)	30 ± 1.0 ^a^	311.48 ± 33.78 ^a^	Colony diameter (mm)	DON synthesis (µg/kg)
1X YPDA	8 ± 0.6 ^c^	218.09 ± 29.52 ^b^	30 ± 0.7	331.00 ± 41.09
0.5X YPDA	20 ± 0.8 ^b^	203.04 ± 8.69 ^b^	32 ± 0.6	340.44 ± 35.00
0.1X YPDA	28 ± 0.7 ^a^	164.83 ± 19.22 ^c^	29 ± 0.6	312.32 ± 30.33

Yeast cells were inoculated on three different media concentrations, and plates from each media, showing almost the same number of yeast CFU were selected for sealing against germinating *F. graminearum* spores. The effect of VOCs released by yeast were tested on fungal growth and mycotoxin synthesis. The values, in each column, followed by different superscript letters are statistically different from each other at ^a^ *p* ≤ 0.05, ^b^ *p* ≤ 0.01 or ^c^ *p* ≤ 0.001.
